# The genomes of the aquarium sponges *Tethya wilhelma* and *Tethya minuta* (Porifera: Demospongiae)

**DOI:** 10.12688/f1000research.150836.2

**Published:** 2024-08-01

**Authors:** Gert Wörheide, Warren R. Francis, Fabian Deister, Stefan Krebs, Dirk Erpenbeck, Sergio Vargas

**Affiliations:** 1 Bayerische Staatssammlung für Paläontologie und Geologie, Staatliche Naturwissenschaftliche Sammlungen Bayerns, Munich, Bavaria, 80333, Germany; 2 GeoBio-Center, Ludwig-Maximilians-Universität München, Munich, 80333, Germany; 3 Earth and Environmental Sciences, Paleontology & Geobiology, Ludwig-Maximilians-Universität München, Munich, 80333, Germany; 4 Laboratory for Functional Genome Analysis (LAFUGA), Gene Center, Ludwig-Maximilians-Universität München, Munich, Germany

**Keywords:** Tethya wilhelma, Tethya minuta, Porifera, Demospongiae, genome, model organism

## Abstract

Sponges (Phylum Porifera) are aquatic sessile metazoans found worldwide in marine and freshwater environments. They are significant in the animal tree of life as one of the earliest-branching metazoan lineages and as filter feeders play crucial ecological roles, particularly in coral reefs, but are susceptible to the effects of climate change. In the face of the current biodiversity crisis, genomic data is crucial for species conservation efforts and predicting their evolutionary potential in response to environmental changes. However, there is a limited availability of culturable sponge species with annotated high-quality genomes to further comprehensive insights into animal evolution, function, and their response to the ongoing global change. Despite the publication of a few high-quality annotated sponge genomes, there remains a gap in resources for culturable sponge species. To address this gap, we provide high quality draft genomes of the two congeneric aquarium species *Tethya wilhelma* and *Tethya minuta*, small ball-shaped demosponges that are easily maintained long-term in *ex situ* culture. As such, they offer promising opportunities as laboratory models to contribute to advancing our understanding of sponge biology and provide valuable resources for studying animal evolution, function, and responses to environmental challenges.

## Introduction

Sponges (Phylum Porifera) are sessile aquatic metazoans that occur globally in marine and freshwater habitats. More than 9,600 valid species have been described, the majority of which (7,989 species) belong to Class Demospongiae. ^1^ Sponges hold a pivotal position in the animal tree of life as one of the earliest metazoan branching lineages, likely originating more than 650 million years ago, ^2^ but their exact phylogenetic position is still disputed. ^3^^–^^6^ As filter feeders, sponges are ecologically important, especially in coral reefs, ^7^^,^^8^ but are also impacted by climate change, as they bleach due to elevated seawater temperatures. ^9^^,^^10^ In the current biodiversity crisis, genome data is valuable to aid species conservation ^11^ and genomic data can be used not only to understand evolution and development, ^12^ but also to predict a species evolutionary potential to adapt to changing environmental conditions due to climate change. ^13^ To improve the understanding of their response to changing environmental conditions, the availability of culturable sponge species with annotated high-quality genomes is important, but only a few sponge species meet both these criteria yet.

Although the first sponge genome was published in 2010 from the Australian demosponge *Amphimedon queenslandica,* ^14^ only a handful of annotated high-quality sponge genomes have been published and analysed since then, for example from the freshwater demosponge *Ephydatia muelleri* ^15^ or the reef-building glass sponge (Class Hexactinellida) *Aphrocallistes vastus.* ^16^ However, only *A. queenslandica* and *E. muelleri* are culturable yet under controlled conditions. This lack of high-quality genome resources available from culturable sponges hinders a full appreciation of our understanding of animal evolution and function as well as the response of sponges as ecological key players in many aquatic ecosystems to the current climate crisis.

To contribute to filling this gap, we here provide high-quality draft genomes of the two aquarium species *Tethya wilhelma* and *Tethya minuta.* These two congeners are small ball-shaped demosponges that were described in 2001 from public aquaria in Germany. ^17^ Due to their long-term culturability, they are a laboratory model for many topics, including multicellularity, early-animal evolution, biomineralization, and even cancer (e.g., Refs. 18– 25). With the provision of novel high-quality genome data of these two species we aim to enhance the use of these species as valuable sponge model systems.

## Methods

### Specimens sampled

Specimens of *Tethya wilhelma* and *Tethya minuta* were obtained from the marine research aquaria of the Chair of Paleontology and Geobiology of the Department of Earth and Environmental Sciences at the Ludwig-Maximilians-Universität München (Germany), where they are cultured since about 2010. No permits were needed for the sampling and processing. Voucher specimens are deposited in the Bavarian State Collection for Paleontology and Geology (SNSB-BSPG) under accession numbers SNSB-BSPG.GW33333 ( *T. wilhelma*) and SNSB-BSPG.GW41624 ( *T. minuta*).

### DNA extraction

For both species, genomic DNA was extracted from either fresh or frozen tissue with a modified cetyltrimethylammonium bromide (CTAB; Carl Roth, Germany, Cat. Nr. 9161.1) extraction. The modification concerned the addition of Potassium acetate (KOAc, Sigma-Aldrich, Germany, Cat. Nr. 791733) in step no. 5 of the protocol. ^26^ Short DNA fragments were removed using AMPure XP (Beckman Coulter, USA, Cat. Nr. A63881) beads to select for long DNA fragments. DNA quantity and quality were controlled on a Nanodrop 1100 and using 1.5% agarose (Biozym, Germany, Cat. Nr. 840004) gels before library preparation as required for the different sequencing platforms used.

### Genome assembly: *Tethya wilhelma*

For *Tethya wilhelma*, we took the genome draft ( *T. wilhelma*-v1) published by our group in 2017 ^20^ as a starting point and used new sequence data and bioinformatics to further improve it. Additional data was obtained using Hi-C ^27^ and Chicago (Dovetail Genomics ^28^) libraries. For this, four whole sponges were frozen in liquid nitrogen and shipped to Dovetail Genomics (Scotts Valley, CA) for library preparation and sequencing. The resulting Chicago/Hi-C reads were processed using Dovetail’s proprietary software HiRise. ^28^ After Chicago/Hi-C scaffolding, the assembly (dubbed *T. wilhelma*-v2) had 1,353 scaffolds, totaling 139 Mb, with an N50 of 5.5 Mb.

While some chromosome-sized scaffolds were evident in *T. wilhelma*’s-v2 assembly, many putative chromosomes remained fragmented. Therefore, we tried to improve the assembly’s contiguity by adding Moleculo long reads as well as Nanopore long reads, the latter derived from a single run of an Oxford Nanopore MinION (see Table 1). The data was assembled using the programs “SSPACE_Long_Read v1-1” ^29^ and “GapCloser v.1.12”. ^30^ This assembly version, called Twi-v3, contained 967 scaffolds, totaling 138.92 Mb, with an N50 of 6.1 Mb (see Table 2).

**Table 1.** Sequencing libraries of *Tethya wilhelma* and *Tethya minuta*. All data can be accessed through the European Nucleotide Archive (ENA) https://www.ebi.ac.uk/ena/browser/view/.

Species	Library	Platform	Type	Reads	Total bp	Accession
*T. wilhelma*	GW33333	MiniSeq	RNA-paired	28.8 M	4.4 Gb	ERR10048047
*T. wilhelma*	Twa2013	Illumina HiSeq 1500	RNA-paired strand specific	100.7 M	25.2 Gb	SRR4255675
*T. wilhelma*	Twa03-2014-02-03	Illumina HiSeq 2000	DNA-paired	129.8 M	26 Gb	SRR2163223
*T. wilhelma*	Tethya_MP	Illumina HiSeq 2000	DNA-paired	140.4 M	35.1 Gb	SRR2296844
*T. wilhelma*	tetwilh1	Moleculo	DNA long reads	125,150	436.7 Mb	SRR5369934
*T. wilhelma*	Dovetail	HiC	DNA	421.4 M	126 Gb	ERR12769028
*T. wilhelma*	Dovetail	Chicago	DNA	430.6 M	129 Gb	ERR12769029
*T. wilhelma*	GW33333	Nanopore	DNA long reads	131,953	2.8 Gb	ERR12769349
*T. wilhelma*	GW33333	10X	DNA	388.47 M	5.4 Gb	ERR12771414
*T. minuta*	GW41624	Nanopore PromethION	DNA long reads	6.39 M	12.73 Gb	ERR12771470
*T. minuta*	GW41624	Nanopore MinION	DNA long reads	545,492	2.17 Gb	ERR12771471
*T. minuta*	GW41624	Illumina HiSeq 1500	DNA-paired	42 M	4.2 Gb	ERR12771519
*T. minuta*	GW41624	Illumina HiSeq 1500	RNA-paired	274 M	13.7 Gb	ERR12771518

**Table 2.** *Tethya wilhelma* assembly statistics. The final version without bacterial scaffolds (Twi-v4-nb) is highlighted in bold.

Version	# scaffolds	Total size (Mb)	N50 (Mb)	N gaps (kb)
Twi-v1	5936	125.67	0.073	1516
Twi-v2	1353	139.49	5.5	2252.2
Twi-v3	967	138.92	6.1	1069.9
Twi-v4	891	138.93	6.7	1077.5
**Twi-v4-no_bacteria**	**557**	**126.1**	**6.7**	**1030.7**

For the assembly of *T. wilhelma*’s genome v4, high molecular weight DNA was extracted, and quality was assessed using a Nanodrop 1100. Fragment size was controlled on a 1.5% agarose gel and an Agilent 2200 TapeStation. Libraries for 10X Genomics (Pleasanton, CA) were generated and sequenced at the University of Potsdam, in collaboration with the group of Prof. M. Hofreiter (Evolutionary Adaptive Genomics, University of Potsdam, Germany), on an Illumina Nextseq500. About 390 M reads were obtained ( Table 1) and assembled using the 10X-Genomics software “Supernova 2.1.1”. ^31^ These assembled contigs were then used for scaffolding the *T. wilhelma* v3 assembly using “SSPACE-LongRead v1-1” ^29^ and then with “P_RNA_scaffolder” ^32^ using 100.7 M PE (125 bp) and 237 M RNA reads (25.2 Gb). Finally, we used hicstuff 2.3.0 ^33^ and the Hi-C data available to create a contact map of the *T. wilhelma *assembly. This assembly, called Twi-v4, had 891 scaffolds with a total size of 138.9 Mb and an N50 of 6.7 Mb, which also included bacterial scaffolds (see Table 2 and below).

### Genome assembly: *Tethya minuta*

For the assembly of *Tethya minuta*, DNA of a single specimen of *Tethya minuta* (sample# GW41624) was extracted with CTAB ^26^ and sequenced twice, using Oxford Nanopore PromethION (12.73 Gb of long reads) and MinION ^34^ (2.17 Gb of long reads). Additionally, we Illumina-sequenced 27 Mbp paired-end (100 PE and 150 PE). These data were assembled with wtdbg2, ^35^ and polished using minimap2. ^36^ SSPACE_LongRead ^29^ was used with the available nanopore data to scaffold the assembly. Finally, we used GapCloser 1.12 ^30^ and the available PE reads to close gaps in the assembly which then had a length of 139 Mb and consisted of 1,043 scaffolds (Tmi-v4), but still included bacterial contigs (see Table 3).

**Table 3.** *Tethya minuta* assembly statistics. The final version without bacterial scaffolds (Tmi-v4-bin17) is highlighted in bold.

Version	# scaffolds	Total size (Mb)	N50 (kb)	N gaps (kb)
Tmi-v3	1043	139.0	788.3	887.0
**Tmi-v4-no_bacteria**	**244**	**86.07**	**969.3**	**534.5**

### Identification of bacterial scaffolds

**Figure 1.**
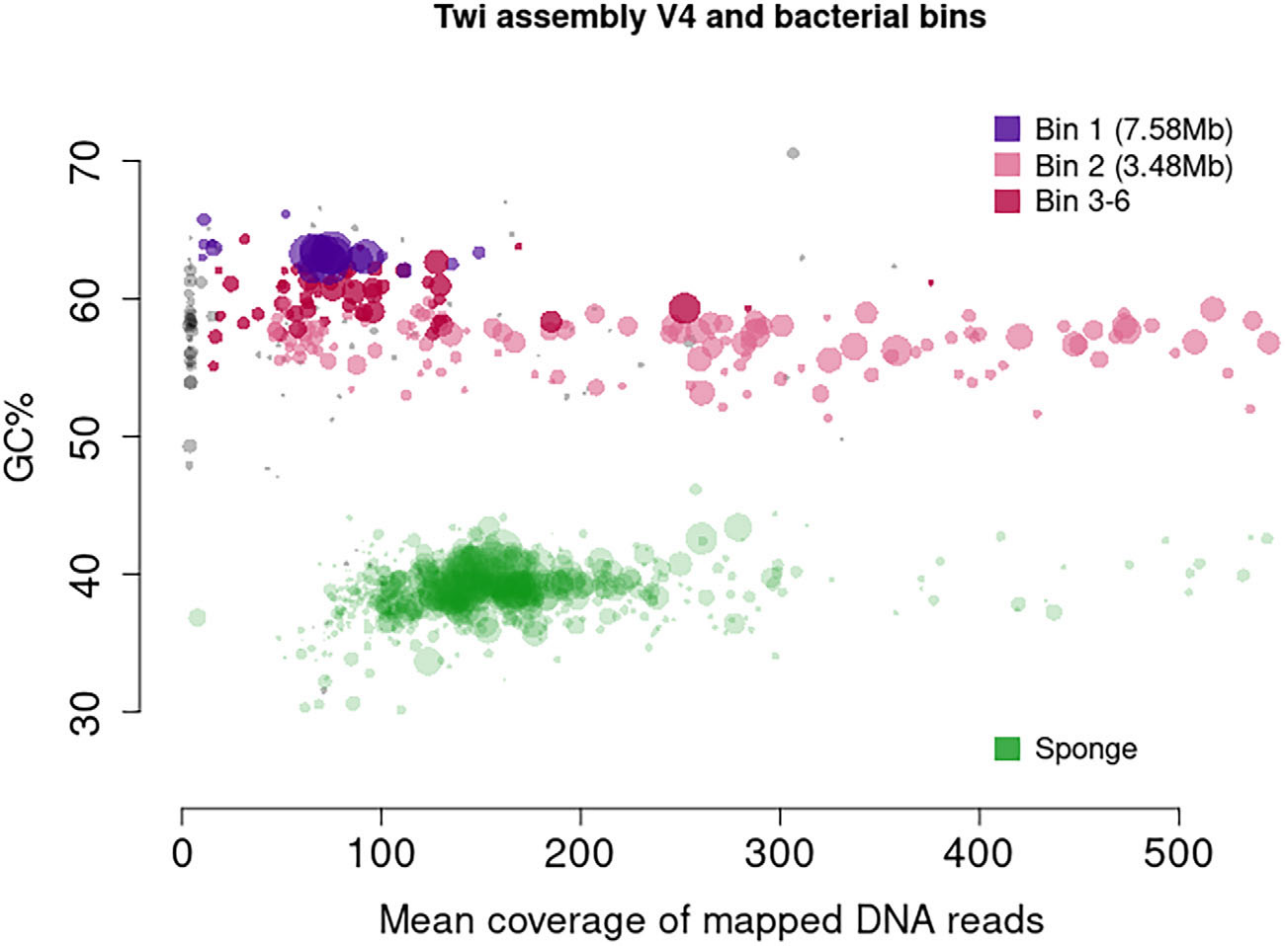
Coverage of mapped DNA reads plotted against GC content of scaffolds of *T. wilhelma* (Twi-v4). MetaBAT v2.15-25 was used for binning. Unidentified scaffolds are shown in grey.

From earlier versions of the genome, it was clear that *Tethya wilhelma* harbours two associated bacteria, both alphaproteobacteria, with an unknown interaction. With the relatively large scaffolds in the assembly, a clear split was seen in GC content and read mapping coverage ( Figure 1). Consequently, we separated all scaffolds with GC content under 47% and defined those as sponge. The remaining scaffolds for the two bacteria were binned using “MetaBAT v2.15-25”, ^37^ with default parameters. For *T. wilhelma*, this yielded 6 bins (see public data repository at Ref. 38 or https://github.com/PalMuc/2Tethya_genomes/tree/main/03-bacteria) with 1 bin corresponding to a single Rhizobiales species (genome appx 7.5 Mb), and the other 5 bins corresponding to a Roseobacter species (genome appx 4.8 Mb). These scaffolds were removed from the final *Tethya wilhelma *genome version. This assembly, called Twi-v4-no_bacteria, had 557 scaffolds with a total size of 126.1 Mb and an N50 of 6.7 Mb ( Table 2; see Ref. 38 or https://github.com/PalMuc/2Tethya_genomes/tree/main/06-FINAL_Assemblies ENA accession GCA_964030475).

**Figure 2.**
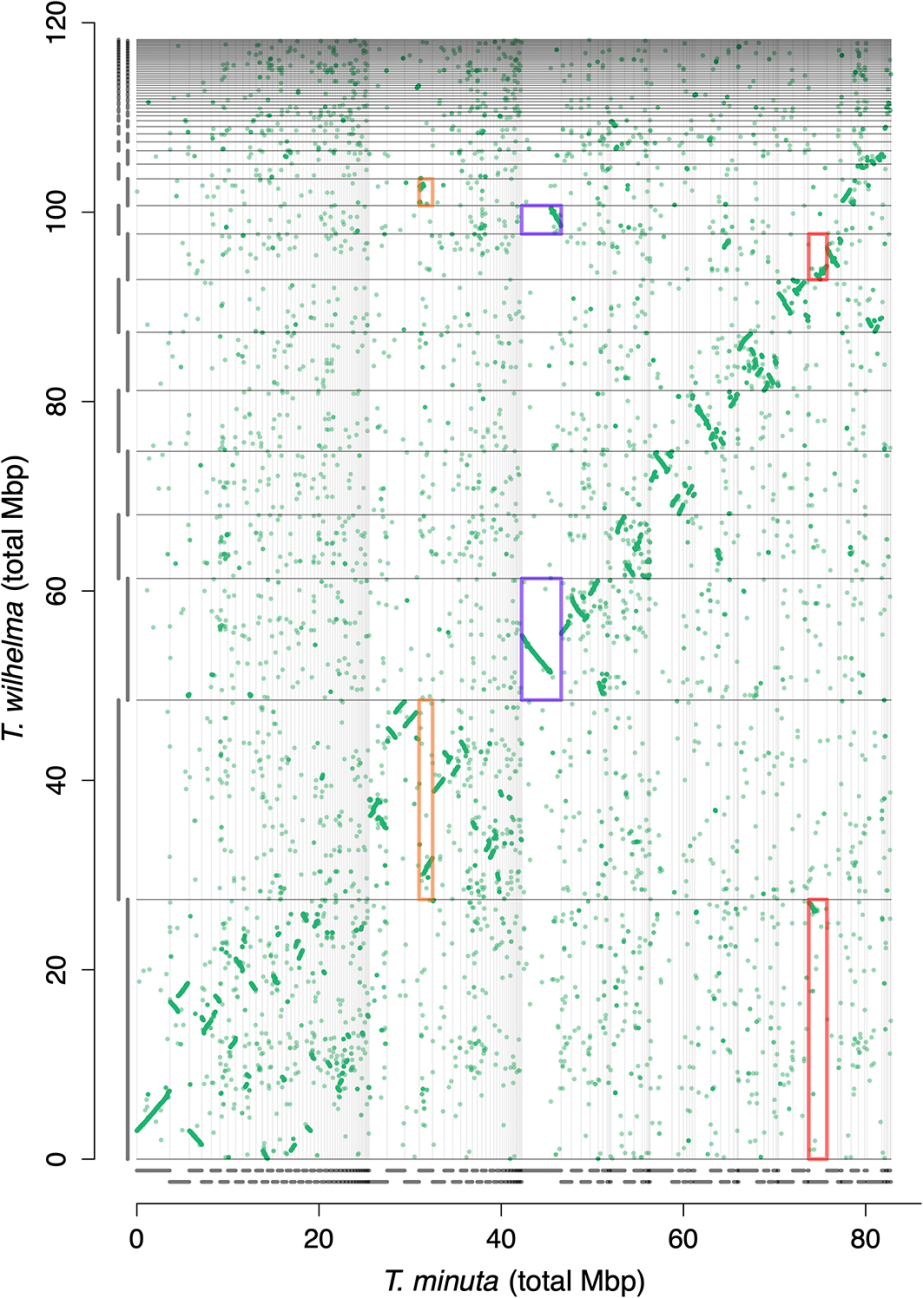
Synteny plot of *Tethya wilhelma* versus *Tethya minuta*. Each point represents a homologous gene between the two species. Bars parallel to each axis show the scaffold size. Scaffolds are arranged from longest to shortest in *T. wilhelma*, with the scaffolds in *T. minuta* sorted to match the *T. wilhelma* scaffold with the most homologs. Several rearrangements are evident, some are shown in the orange, blue, and red boxes, which could result from translocations, inversions on currently incomplete scaffolds, or misassemblies.

For *T. minuta*, we used “MetaBAT v2.15-25” ^37^ to identify and separate bacterial contigs from sponge scaffolds, which had produced 23 bins from the assembly. One of the bins, numbered as bin-17, contained the bulk of the assembly, and was identified as originating from the sponge due to the GC content of 38.3% and substantial RNAseq mapping. This bin (here now called Tmi-v4-no_bacteria, Table 3) had 244 scaffolds with a total size of 86.07 Mb, around 40 Mb smaller than the assembly of *T. wilhelma* ( Table 2, see Ref. 38 or https://github.com/PalMuc/2Tethya_genomes/tree/main/06-FINAL_Assemblies; ENA accession GCA_964030485). Nearly all of the large chromosomal pieces in *T. wilhelma* had matching pieces among the scaffolds of *T. minuta*, as evident on the synteny plot ( Figure 2), which suggested that the assembly of *T. minuta* was smaller not because of missing scaffolds or mis-assemblies, but merely from a smaller genome.

### RNA extraction, sequencing, and assembly

RNA was extracted from fresh tissue of *Tethya minuta* using TRIzol (Fisher Scientific, Germany, Cat. Nr. 12034977) and chloroform (Carl Roth, Germany, Cat. Nr. 3313.1) precipitation ^39^ with subsequent quality control on a Bioanalyzer 2100. Libraries were prepared and sequenced twice using one third of a lane of an Illumina HiSeq1500 (100 bp and 50 bp) at the LMU GeneCenter, yielding 137 M read pairs. Reads were assembled *de novo* using Trinity, ^40^ using default parameters, resulting in an assembly of 151,079 contigs with an average length of 677 bp. This assembly was also used as a training set for *de novo* gene prediction (see below). Transcriptome sequencing and assembly of *Tethya wilhelma* has been described in Francis et al. ^20^ Statistics of the different sequencing libraries of *Tethya wilhelma* and *Tethya minuta* are given in Table 1.

### Gene annotation

Both *Tethya* species were annotated using AUGUSTUS. For *T. wilhelma*, we used the BRAKER v2.0 pipeline, ^41^ with the options --useexisting --species=Tethya_wilh and including mapped RNA. This predicted a total of 28,113 gene models, which were used for downstream analysis.

For *Tethya minuta*, the assembly Tmi-v4-no_bacteria and the *de novo* Trinity assembly were used as inputs for WebAUGUSTUS. ^42^ This yielded 22,779 gene models and 33,041 genes (see files ‘hints_pred’ and ‘hints_UTR_pred’ at Ref. 38 or https://github.com/PalMuc/2Tethya_genomes/tree/main/05-annotation/tethya_minuta_augustus).

## Results

The final version of the *Tethya wilhelma* draft genome assembly (see Table 2, Twi-v4-no_bacteria) without bacterial scaffolds has 557 scaffolds, a length of 126.1 MB, an N50 of 6.7 MB, and contains 1030.7 kb gaps (Ns). The final version of the *Tethya minuta* draft genome assembly (see Table 3, Tmi-v4-no_bacteria) without bacterial scaffolds has 244 scaffolds, a length of 86.07 MB, an N50 of 969.3 kb, and contains 534.5 kb gaps (Ns). BUSCO values for the two assemblies are given in Table 4.

**Table 4.** *Tethya wilhelma* (Twi-v4) and *Tethya minuta* (Tmi-v4) (assemblies without bacteria) BUSCO-values for lineage dataset metazoa_odb10.

BUSCO results	Twi-v4-no_bacteria	Tmi-v4-no_bacteria
Total query BUSCOs	954	954
Complete BUSCOs (C)	800 (83.6%)	552 (57.9%)
Complete and single-copy (S)	776 (81.3%)	498 (52.2%)
Complete and duplicated (D)	24 (2.5%)	54 (5.7%)
Fragmented (F)	63 (6.6%)	186 (19.5%)
Missing (M)	91 (9.5%)	216 (22.6%)

### Ethical considerations

For work with sponges (Porifera) no ethical clearing is needed.

## Data Availability

Raw reads are available from the European Nucleotide Archive under bioproject numbers PRJNA288690, PRJEB53671, for individual accession numbers see Table 1. The assembled genomes are also available in the European Nucleotide Archive: *Tethya wilhelma* GCA_964030475, *Tethya minuta* GCA_964030485. Further data on the genome assemblies, including analytical pipelines and scripts, is available in the public repository https://github.com/PalMuc/2Tethya_genomes, archived at Zenodo ( https://zenodo.org/doi/10.5281/zenodo.10991740). ^38^ Data are available under the terms of the Creative Commons Attribution 4.0 International license (CC BY-SA 4.0 DEED) ( https://creativecommons.org/licenses/by-sa/4.0/). Voucher specimens are deposited in the Bavarian State Collection for Paleontology and Geology (SNSB-BSPG, https://bspg.snsb.de) under accession numbers SNSB-BSPG.GW33333 ( *T. wilhelma*) and SNSB-BSPG.GW41624 ( *T. minuta*).
