# Supplementary material for: The genomes of the aquarium sponges Tethya wilhelma and Tethya minuta (Porifera: Demospongiae)
Source: F1000Res. 2024 Aug 1;13:679. Originally published 2024 Jun 24. [Version 2] doi: 10.12688/f1000research.150836.2 (PMC11347921; doi:10.12688/f1000research.150836.2)
Supplement: Peer review history

# Reviewer report: Reviewer response for version 2

**DOI:** 10.5256/f1000research.169477.r309948

**Published:** 2024-08-26

**Authors:** Bernie Degnan

**Affiliations:** 1 School of the Environment, University of Queensland, Queensland, Australia

The responses and the revision looks fine.  The revised figure is greatly improved.

Are the datasets clearly presented in a usable and accessible format, and the assembly and annotation available in an appropriate subject-specific repository?

Partly

Are sufficient details of the sequencing and extraction, software used, and materials provided to allow replication by others?

Partly

Are the rationale for sequencing the genome and the species significance clearly described?

Yes

Are the protocols appropriate and is the work technically sound?

Yes

Reviewer Expertise:

NA

I confirm that I have read this submission and believe that I have an appropriate level of expertise to confirm that it is of an acceptable scientific standard.

# Reviewer report: Reviewer response for version 1

**DOI:** 10.5256/f1000research.165438.r295815

**Published:** 2024-07-13

**Authors:** Eric Bautista-Guerrero

**Affiliations:** 1 Laboratorio de Ecología Marina, Centro de Investigaciones Costeras, Centro Universitario de la Costa, University of Guadalajara, Guadalajara, Jalisco, Mexico

Comments to the Author

 This note represents a significant contribution to the field of sponge biology, advancing our understanding of this organism and providing valuable metagenomic resources. It proposes the use of two species of sponge that can be easily maintained long-term in ex situ culture. The sponges in question are proposed as model systems for studying the responses of this invertebrate to the effects of climate change.

 Furthermore, the sample set is remarkable, and the sequencing and bioinformatics techniques used to improve the draft genome are noteworthy.

 Overall, the Genome note presents a high-quality bioinformatic analysis, and it is recommended that it be accepted pending consideration of minor revisions as detailed below.

 Keywords

 1. First line– Please use italics for the following: Tethya wilhelma, Tethya minuta.

 Methods

 2. DNA extraction: it would be helpful if a brief explanation the modifications to the CTAB method.

 3. In the sentence: “Fragment size was controlled on a 1.5 agarose gel”…., add the percentage should be added after 1.5 (1.5%).

 4. Figure 2: Axes of the graph, scientific name of the sponges in italics.  

Are the datasets clearly presented in a usable and accessible format, and the assembly and annotation available in an appropriate subject-specific repository?

Yes

Are sufficient details of the sequencing and extraction, software used, and materials provided to allow replication by others?

Yes

Are the rationale for sequencing the genome and the species significance clearly described?

Yes

Are the protocols appropriate and is the work technically sound?

Yes

Reviewer Expertise:

I'm an expert in integrative taxonomy and molecular phylogeny of marine sponges. I believe that the genome of these two sponges will contribute significantly to our understanding of how this group of invertebrates responds to climate change.

I confirm that I have read this submission and believe that I have an appropriate level of expertise to confirm that it is of an acceptable scientific standard.

# Response

**Published:** 2024-07-28

**Authors:** Gert Wörheide

**Affiliations:** Earth and Environmental Scienc, Ludwig-Maximilians-Universität München, Munich, Bavaria, Germany

Dear Professor Bautista-Guerrero,

 Thank you very much for your constructive comments on our manuscript to which we respond point by point below:

**1. First line (Keywords) – Please use italics for the following: Tethya wilhelma, Tethya minuta.**

***Authors reply:*** Thanks, correct. However, in the Word File I downloaded for the revision the species names in the keywords are correctly formatted in italics, so this must be a typesetting issue on publisher’s side. I will alert them to the fact.

**2. DNA extraction: it would be helpful if a brief explanation the modifications to the CTAB method.**

***Author’s reply:*** Thank for this comment, although the modifications are detailed in the protocol (citation 26) at protocols.io we have now included the following sentence:

 The modification concerned the addition of Potassium acetate (KOAc, Sigma-Aldrich, Germany, Cat. Nr. 791733) in step no. 5 of the protocol ^26^.

**3. In the sentence: “Fragment size was controlled on a 1.5 agarose gel”…., add the percentage should be added after 1.5 (1.5%).**

***Author’s reply: *** Thanks for picking this up, we have added the % after 1.5

**4. Figure 2: Axes of the graph, scientific name of the sponges in italics.**

***Author’s reply: *** Thanks, and sorry for this oversight, this has now been corrected in the revised Figure 2 (see reply to comment 3 by Bernie Degnan (Reviewer 1).

# Reviewer report: Reviewer response for version 1

**DOI:** 10.5256/f1000research.165438.r295816

**Published:** 2024-07-12

**Authors:** Bernie Degnan

**Affiliations:** 1 School of the Environment, University of Queensland, Queensland, Australia

Woerheide et al. provide a strong rationale for the sequencing, assembly and analysis of the genomes of two congeneric demosponges, *Tethya wilhelma* and *T. minuta, *both which are readily found in public marine aquaria. Using a combination long and short read sequencing and Dovetail HiC/Chicago sequencing, the authors substantially improved the original *T. wilhelma* genome assembly. The *T. minuta *assembly, based on long and short reads only, is of sufficient quality for comparison with *T. wilhelma* genome.

 Suggested revisions: 1.The authors should include details about how the sponge DNA was procured by Dovetail for HiC/Chicago as this could be useful for the sequencing of other sponge and marine invertebrate genomes. 2. Several of the accession numbers in Table 1 can not be traced in NCBI - please update.  3. The synteny plot comparing the two *Tethya* genomes (Fig. 2) is difficult to interpret and should be improved/reordered to allow better direct comparison of congener scaffolds.

Are the datasets clearly presented in a usable and accessible format, and the assembly and annotation available in an appropriate subject-specific repository?

Partly

Are sufficient details of the sequencing and extraction, software used, and materials provided to allow replication by others?

Partly

Are the rationale for sequencing the genome and the species significance clearly described?

Yes

Are the protocols appropriate and is the work technically sound?

Yes

Reviewer Expertise:

Marine biology, sponges, genomes, transcriptomes

I confirm that I have read this submission and believe that I have an appropriate level of expertise to confirm that it is of an acceptable scientific standard, however I have significant reservations, as outlined above.

# Response

**Published:** 2024-07-28

**Authors:** Gert Wörheide

**Affiliations:** Earth and Environmental Scienc, Ludwig-Maximilians-Universität München, Munich, Bavaria, Germany

Dear Professor Degnan,

 thank you very much for your constructive comments on our manuscript to which we respond point by point below:

**1. The authors should include details about how the sponge DNA was procured by Dovetail for HiC/Chicago as this could be useful for the sequencing of other sponge and marine invertebrate genomes.**

***Authors reply:*** Unfortunately, we do not have information how DNA was procured by Dovetail. As stated, we sent them four whole sponges and they extracted chromatin for Chicago and Hi-C according to their proprietary protocols. We then only received the data, without any information on wet-bench protocols. We contacted Dovetail in this matter but have yet to receive a reply.

**2. Several of the accession numbers in Table 1 can not be traced in NCBI - please update.**

***Authors reply:*** The accession number can all be traced in the European Nucleotide Archive (ENA), to which the data was submitted (we double checked all entries). We have, unfortunately, no influence on the timing of data exchange between partners in the International Nucleotide Sequence Database Collaboration (INSDC), where both ENA and NCBI participate.

 We have now included the direct link to the ENA browser to ease access. https://www.ebi.ac.uk/ena/browser/view/

**3. The synteny plot comparing the two Tethya genomes (Fig. 2) is difficult to interpret and should be improved/reordered to allow better direct comparison of congener scaffolds.**

***Authors reply: *** Thank you very much for this great suggestion, we have now reordered the plot and highlight some rearrangements in boxes in the plot. We also now provide a much more detailed explanation in the figure caption, i.e.,

 Figure 2: Synteny plot of *Tethya wilhelma* versus *Tethya minuta*. Each point represents a homologous gene between the two species. Bars parallel to each axis show the scaffold size. Scaffolds are arranged from longest to shortest in *T. wilhelma*, with the scaffolds in *T. minuta* sorted to match the *T. wilhelma* scaffold with the most homologs. Several rearrangements are evident, some are shown in the orange, blue, and red boxes, which could result from translocations, inversions on currently incomplete scaffolds, or misassemblies.
